# Checkpoint on Adrenal Insufficiency: Optimizing Screening in Immune Checkpoint Inhibitor Therapy

**DOI:** 10.7759/cureus.91730

**Published:** 2025-09-06

**Authors:** Hima Darapu, Navya Konindala, Ravi Paluri

**Affiliations:** 1 Endocrinology, Internal Medicine, Wake Forest University School of Medicine, Winston Salem, USA; 2 Endocrinology, Diabetes and Metabolism, Atrium Health Wake Forest Baptist Medical Center, Winston Salem, USA; 3 Hematology and Oncology, Atrium Health Wake Forest Baptist Medical Center, Winston Salem, USA

**Keywords:** adrenal insufficiency (ai), checkpoint inhibitor therapy, ctla-4 inhibitor, endocrine irae, hypophysitis immune checkpoint inhibitors (icis), immune checkpoint inhibitor-induced endocrinopathies, immune-related adverse event (irae), ipilimumab, ipilimumab-related adverse events

## Abstract

Background

Differentiating immune checkpoint inhibitor (ICI)-induced endocrinopathies from cancer-related symptoms or other treatment toxicities is challenging. Biochemical surveillance is essential, especially in patients with new or worsening fatigue and weakness. Current professional society guidelines differ on recommendations for baseline and follow-up biochemical screening for adrenal insufficiency (AI ), and real-world adherence to these protocols remains uncertain. This study evaluates current screening practices for AI in patients receiving ipilimumab-nivolumab combination therapy (a CTLA-4-containing regimen), quantifies the incidence of AI, and identifies gaps in early detection of these endocrinopathies.

Methods

We conducted a retrospective cohort study of adult patients who received ipilimumab either as monotherapy or in combination with nivolumab at a tertiary academic hospital between June 1, 2023, and June 30, 2024. Data were extracted from the Epic electronic health record and included demographics, cancer type and stage, ICI regimen, and laboratory values, including 8:00 AM cortisol, adrenocorticotropic hormone (ACTH), and thyroid-stimulating hormone (TSH) obtained at baseline and during follow-up. The primary outcome was the proportion of patients screened for adrenal insufficiency during therapy. Secondary outcomes included the incidence of secondary AI, screening rates for ICI-associated thyroid dysfunction, and timing and clinical setting of AI diagnosis.

Results

A total of 185 patients received ipilimumab-nivolumab during the study period. Only one patient underwent both baseline and interval ACTH and cortisol measurements. All patients had baseline TSH levels, checked prior to starting immunotherapy, and follow-up TSH levels were measured at every treatment cycle. Secondary AI was diagnosed in 17 patients (9.1%). Diagnosis occurred in six patients (35.3%) during inpatient hospitalization, three (17.6%) in the emergency department, and eight (47.1%) in the outpatient setting. The most common presenting symptom was fatigue (35.3%), followed by nausea (29.4%) and vomiting (23.5%).

Conclusion

Adrenal insufficiency secondary to ICI-induced hypophysitis remains under-recognized in patients receiving ipilimumab-nivolumab combination therapy, with most cases identified only after symptom onset in acute care settings. While thyroid function was consistently monitored, adrenal insufficiency screening was rarely performed. Standardized protocols that include baseline and periodic ACTH and cortisol testing are needed to enable earlier detection and intervention, potentially reducing morbidity, hospitalizations, and healthcare utilization in this high-risk population.

## Introduction

Immune checkpoint inhibitors (ICIs) have revolutionized the treatment of multiple cancers and are now approved for diverse malignancies, including melanoma, non-small cell lung cancer, and renal cell carcinoma [1]. Despite their substantial therapeutic benefit, ICIs are associated with a broad range of immune-related adverse events (irAEs) that can affect nearly every organ system [1]. The gastrointestinal, dermatologic, and endocrine systems are among the most frequently involved, with endocrinopathies representing particularly serious complications because they can be life-threatening if not promptly recognized and managed [1].

Hypophysitis is a well-recognized endocrine irAE, characterized by pituitary inflammation, resulting in secondary adrenal insufficiency (AI), central hypothyroidism, and sometimes hypogonadotropic hypogonadism [1]. Its incidence ranges from <1% with PD-1/PD-L1 inhibitors to as high as 6-17% with CTLA-4 blockade, particularly in combination regimens [2]. Clinical manifestations, including fatigue, nausea, and generalized weakness, are nonspecific and frequently mistaken for cancer progression or chemotherapy-related toxicities. This overlap often delays diagnosis and treatment. Managing endocrine irAEs remains an increasing clinical challenge, and several professional societies have issued guidelines for their evaluation and management [3-7]. Nonetheless, real-world adherence to standardized screening and monitoring remains limited.

The mechanisms underlying ICI-induced endocrine irAEs are incompletely understood. ICIs are thought to disrupt immune tolerance and promote T-cell activation against self-antigens within endocrine tissues. CTLA-4 expression has been demonstrated on pituitary cells, and PD-L1 expression has been identified in the thyroid, pituitary, and pancreas, suggesting that these sites may be particularly vulnerable to T-cell-mediated cytotoxicity during ICI therapy [8]. This may account for the occurrence of hypophysitis, thyroiditis, and insulin-deficient diabetes.

Epidemiologic data highlight the burden of hypophysitis in patients on ICI therapy. A meta-analysis of 34 studies, including 6,472 patients, reported hypophysitis in 85 cases, with a 6.4% incidence among those receiving combination anti-CTLA-4 and anti-PD-1 therapy [9]. Other large retrospective cohorts have described even higher rates, ranging from 6.7% to 13% [10,11]. These findings demonstrate the importance of improved surveillance strategies in high-risk patients. CTLA-4 blockade, either alone or in combination with PD-1/PD-L1 inhibitors, has been linked to dose-dependent immune toxicities, with the incidence and severity of irAEs generally increasing at higher doses [12,13]. Patients with preexisting autoimmune disorders or baseline organ dysfunction appear particularly vulnerable [14].

Given that hypophysitis is more frequently observed with CTLA-4 inhibitors than with PD-1/PD-L1 inhibitors and occurs at even higher rates with dual combination regimens, the present study focuses on patients receiving ipilimumab-nivolumab therapy, a CTLA-4-containing regimen associated with an elevated risk of hypophysitis. The objective is to evaluate current screening practices for secondary AI in this high-risk population and to identify critical gaps in early detection and management.

## Materials and methods

Study design and setting

This retrospective cohort study was conducted at a tertiary academic medical center and included patients treated between June 1, 2023, and June 30, 2024. The study was approved by the institutional review board (IRB), and all data were handled in accordance with institutional policies on patient confidentiality.

Study population

Eligible participants were adults (≥18 years) who received ipilimumab either as monotherapy or in combination with nivolumab for any cancer type during the study period. Patients were excluded if they had received PD-1 inhibitor monotherapy or any CTLA-4 inhibitor other than ipilimumab, to ensure a homogeneous cohort and because ipilimumab is the predominant CTLA-4 inhibitor used at our institution.

Data collection

Data were retrospectively collected through a comprehensive review of patient medical records using the Epic 3 electronic health record (EHR) system. Extracted variables included demographic information (age, sex, and ethnicity), cancer type and stage, and details of the immune checkpoint inhibitor regimen, including start and end dates. Laboratory data comprised baseline measurements performed within 30 days prior to ICI initiation and follow-up measurements obtained during treatment or within 90 days of the final dose. Specifically, results for 8:00 AM serum cortisol, adrenocorticotropic hormone (ACTH), and thyroid-stimulating hormone (TSH) were collected for analysis. Clinical outcome variables focused on secondary adrenal insufficiency and included its incidence, timing of diagnosis, clinical setting at the time of diagnosis (hospitalization, emergency department, or outpatient visit), and presenting symptoms. All data were de-identified prior to analysis to maintain patient confidentiality.

Laboratory procedures

All hormone assays were performed in the Wake Forest Clinical Chemistry Laboratory. Serum cortisol and ACTH were measured using the Roche Elecsys electrochemiluminescence immunoassay platform. The reference range for serum cortisol at 8:00 AM was 6.0-18.4 µg/dL, and for ACTH was 7.2-63 pg/mL. TSH was measured using the same platform, with a reference range of 0.45-4.5 µIU/mL.

Data analysis 

Data were summarized using descriptive statistics. Patient demographics, cancer characteristics, and treatment regimens were presented as counts and percentages. Laboratory screening practices for adrenal and thyroid function were reported as proportions of the total study population. The incidence of adrenal insufficiency was calculated as the number of confirmed cases divided by the total cohort. Clinical presentation and diagnostic setting of adrenal insufficiency (inpatient, emergency department, or outpatient) were tabulated as frequencies and percentages. No inferential statistical testing was performed, as the study objective was to describe screening practices and the incidence of adrenal insufficiency in this cohort.

Outcomes

The primary outcome was the proportion of patients who underwent baseline and follow-up screening for adrenal insufficiency. This directly reflects our central objective of evaluating real-world adherence to recommended endocrine surveillance practices in patients receiving ipilimumab-nivolumab therapy.

The secondary outcomes were selected to provide a clinical context and assess the broader implications of screening practices. These included the following: (1) the incidence of secondary AI in the study cohort, which helps quantify the clinical burden of this complication; (2) screening rates for ICI-associated thyroid dysfunction, included for comparison since thyroid monitoring is more consistently embedded in oncology protocols; and (3) the timing and clinical setting of AI diagnosis (inpatient, emergency department, or outpatient), to evaluate where missed or delayed detection of AI most commonly occurs.

Adrenal insufficiency was diagnosed according to standard guideline-based criteria, using established cortisol and ACTH cutoffs, though the present study primarily focused on screening practices rather than the diagnostic process itself.

## Results

A total of 185 patients received ipilimumab-nivolumab therapy between June 1, 2023, and June 30, 2024 (Table 1). Only one patient (0.5%) underwent serial screening for adrenal insufficiency with both baseline and follow-up ACTH and morning cortisol measurements. In contrast, thyroid function was consistently monitored, with all patients undergoing baseline TSH testing prior to treatment initiation and follow-up TSH testing at every treatment cycle.

**Table 1:** Baseline characteristics and screening practices in patients receiving ipilimumab-based therapy The table summarizes the baseline demographic and oncologic characteristics of the study cohort, along with endocrine screening practices prior to and during immune checkpoint inhibitor (ICI) therapy. Abbreviations: ACTH – adrenocorticotropic hormone; CTLA-4 – cytotoxic T-lymphocyte-associated protein 4; ICI – immune checkpoint inhibitor; NSCLC – non-small cell lung carcinoma; TSH – thyroid-stimulating hormone; RCC – renal cell carcinoma.

Variable	Number of Patients (n)
Total study participants	185
Age, mean ± SD (years)	63.0 ± 11.7
Gender	Male: 120
	Female: 65
Ethinicity	Asian: 22
	African American: 7
	Caucasian: 149
	American Indian: 2
	Unknown: 5
Primary cancer diagnosis	Melanoma: 69/185
	RCC: 27/185
	NSCLC: 53/185
	Other: 36/185
CTLA-4–based ICI regimen	Ipilimumab monotherapy: 0/185
	Ipilimumab + nivolumab: 185/185
Baseline labs prior to starting ICI	TSH: 185/185
	AM cortisol: 8/185
	ACTH: 4/185
Screening labs prior to each ICI	TSH: 185/185
	Cortisol and ACTH follow-up: 1/185

Secondary adrenal insufficiency was diagnosed in 17 patients, yielding an incidence of 9.1%. The timing and setting of diagnosis varied: six cases (35.3%) were identified during hospitalization, three (17.6%) in the emergency department, and eight (47.1%) in the outpatient setting. The most common presenting symptom was fatigue (35.3%), followed by nausea (29.4%) and vomiting (23.5%) (Table 2).

**Table 2:** Adrenal insufficiency screening patterns and outcomes in ipilimumab-treated patients The table summarizes secondary adrenal insufficiency outcomes in patients receiving ipilimumab-based therapy, including incidence, screening frequency, timing, setting of AI diagnosis, and presenting symptoms. Values are presented as counts unless otherwise specified. Abbreviations: ACTH – adrenocorticotropic hormone; AI – adrenal insufficiency; irAEs – immune-related adverse events; ICI – immune checkpoint inhibitor; ED – emergency department.

Variable	Number of patients (n)
Patients diagnosed with adrenal insufficiency	17/185
Serial Cortisol/ACTH screening prior to each ICI cycle	1/185
Timing of diagnosis	Inpatient: 6/17
	ED: 3/17
	Outpatient: 9/17
Presenting symptoms prior to AI diagnosis	Fatigue: 6/17
	Nausea: 5/17
	Vomiting: 4/17

## Discussion

Differentiating ICI-induced endocrinopathies from cancer-related symptoms or treatment-related side effects, particularly those of chemotherapy, can be challenging, especially as combination regimens become more common. This overlap in presentation can obscure suspicion of adrenal insufficiency. Biochemical surveillance is therefore critical, particularly in patients with new or worsening fatigue, weakness, or nonspecific symptoms, such as nausea or vomiting. Current recommendations vary: ESE advises a baseline 8:00 AM cortisol before starting ICI therapy, followed by testing every four to six weeks [6], while the American Association of Clinical Endocrinology (AACE) recommends baseline ACTH and cortisol measurements with subsequent monitoring guided by clinical presentation [7].

Delayed diagnosis is common due to nonspecific symptoms that may be attributed to the malignancy or treatment toxicity. Such delays can result in preventable complications, including higher morbidity, prolonged hospital stays, and increased healthcare costs. A US claims database review found that 3.5% of ICI-treated patients experienced severe irAEs requiring hospitalization, and those patients were more likely to be rehospitalized, have higher in-hospital mortality, and be less likely to resume ICI therapy [15,16].

Our study reveals a significant gap in adrenal insufficiency screening among patients receiving ipilimumab-nivolumab combination regimen, reflecting gaps in endocrine monitoring in CTLA-4 containing therapies. Although all patients were appropriately screened for thyroid dysfunction, only one of 185 underwent both baseline and interval ACTH and cortisol testing. Among the 17 patients diagnosed with adrenal insufficiency, most cases were identified in acute care settings, such as the emergency department or during hospitalization, rather than during routine follow-up. More than half required inpatient care, emphasizing the clinical impact of delayed recognition. These findings call attention to the need for standardized endocrine monitoring protocols, including baseline and periodic ACTH and cortisol measurements in high-risk patients.

Practical barriers to routine adrenal screening include the difficulty of coordinating morning cortisol testing in busy oncology clinics and the challenge of interpreting results in the context of recent corticosteroid use, which can suppress endogenous cortisol. Addressing these issues may involve careful timing relative to the last steroid dose, consideration of steroid half-life, and early involvement of endocrinology when results are equivocal. In contrast, thyroid function testing is consistently performed because TSH measurement is straightforward, not time-dependent, and firmly embedded in standard oncology protocols. This difference explains why adrenal surveillance lags despite the potentially greater morbidity associated with missed adrenal insufficiency. Embedding cortisol and ACTH testing into standardized workflows similar to TSH may help reduce this disparity and promote earlier recognition of adrenal dysfunction.

This study has a few limitations. It was a single-center, retrospective analysis with a relatively small sample size, limiting generalizability. Reliance on electronic medical records and laboratory data may have introduced reporting bias and incomplete capture of external results. Because adrenal insufficiency screening was not standardized during the study period, subclinical or asymptomatic cases may have been missed, leading to an underestimation of the incidence. The focus on ipilimumab-containing regimens, while intentional to maintain a homogeneous cohort, limits applicability to PD-1/PD-L1 monotherapies, which have a lower reported incidence of hypophysitis [17].

In summary, although endocrine irAEs are increasingly recognized in the era of immunotherapy, our study demonstrates that adrenal insufficiency remains under-screened, under-diagnosed, and often identified only after significant delays in real-world oncology practice. Implementing standardized biochemical surveillance can enable earlier detection, reduce morbidity, decrease hospitalizations, and ultimately improve patient outcomes. Strong collaboration between oncology and endocrinology teams is critical to establishing effective monitoring protocols and ensuring comprehensive care for patients receiving immune checkpoint inhibitors.

Future research should focus on prospective studies or pragmatic clinical trials to evaluate whether implementation of standardized endocrine screening protocols improves early detection, reduces morbidity, and lowers healthcare utilization in patients receiving CTLA-4-containing regimens.

## Conclusions

Adrenal insufficiency due to ICI-induced hypophysitis, particularly in the setting of ipilimumab-nivolumab combination therapy (CTLA-4-containing regimens), remains an under-recognized yet clinically significant complication. Our study highlights a notable gap in routine endocrine screening, with most cases identified only after the onset of significant symptoms and often requiring acute care intervention. While thyroid function was consistently monitored, surveillance for adrenal insufficiency was infrequent.

These findings highlight the importance of clinician awareness and the need to emphasize proactive screening in all patients receiving CTLA-4-based therapy, especially if on dual therapy with ipilimumab and nivolumab, regardless of symptomatology. This is especially important given the difficulty in distinguishing adrenal insufficiency from the nonspecific symptoms commonly encountered in oncology patients, particularly those undergoing concurrent chemotherapy. Early identification through routine biochemical monitoring in high-risk populations may reduce morbidity, prevent unnecessary hospitalizations, and enhance overall patient care. The adoption of standardized screening protocols, supported by coordinated efforts between oncology and endocrinology teams, is essential to improving outcomes in this vulnerable patient population.
